# Impact of Online Diabetes Education Counseling on Treatment adherence and Quality of Life of Type-II Diabetics

**DOI:** 10.12669/pjms.41.3.10468

**Published:** 2025-03

**Authors:** Ibrar Ahmed, Adan Javed, Nazeer Ullah, Hoor Maab Kaifi

**Affiliations:** 1 Ibrar Ahmed, Assistant Professor, Department of Endocrinology, Lady reading hospital, Peshawar, Pakistan; 2 Adan Javed, Diabetes Educator, Department of Endocrinology, Lady reading hospital, Peshawar, Pakistan; 3 Nazeer Ullah, Diabetes Educator, Department of Endocrinology, Lady reading hospital, Peshawar, Pakistan; 4 Hoor Maab Kaifi, Dietician, Department of Endocrinology, Lady reading hospital, Peshawar, Pakistan

**Keywords:** Drug adherence, Oral anti-diabetic drugs, Random blood sugar, self-care management, Type-II diabetes mellitus

## Abstract

**Objective:**

To determine the impact of online diabetes education counseling on treatment adherence and quality of life of Type-II diabetics in Mardan, Khyber Pakhtunkhwa, Pakistan

**Method:**

This was a quasi-experimental study conducted in Mardan and its peripheries, Khyber Pakhtunkhwa (KPK), Pakistan. The duration of the study was from October 2022 till March 2023. A total of 196 patients with diagnosed Type-II Diabetes mellitus were enrolled in the study using non-probability consecutive sampling technique. Baseline Information was gathered regarding demographics, dietary pattern, medicine adherence, quality of life, Random blood sugar (RBS), HbA1c, Blood pressure, and BMI. Counseling regarding Diabetes treatment and lifestyle were carried out for each patient, patients were followed twice weekly for a period of six months with possible interventions if needed. RBS, HbA1c, Blood pressure, BMI, medicine adherence and quality of life were determined on last visit.

**Results:**

The study showed that out of 196 patients, 108(54.6%) were males and 88(45.4%) were females. Statistically significant difference was observed between baseline and at six months follow up with respect to RBS, HbA1c, BMI and blood pressure, (p<0.001). Drug adherence was significantly improved after intervention (p<0.05).

**Conclusion:**

Online diabetes education counseling had a significant impact on treatment adherence. Post intervention quality of life of Type-II diabetics was adequate. Moreover, online diabetes education counseling significantly reduced the blood glucose level, body mass Index and blood pressure.

## INTRODUCTION

Diabetes mellitus (DM) and its complications are a major cause of early mortality worldwide. In 2019, it was estimated that over four million individuals aged 20 to 79 years died from DM-related complications.^1^ DM significantly affects individuals, their families, social dynamics, and healthcare systems, ultimately reducing quality of life, increasing disability, and worsening chronic conditions.^1^,^2^

In 2019, the World Health Organization (WHO) ranked Pakistan 4th globally, with a diabetic population of 19.4 million, projected to rise to 26.2 million by 2030.^3^,^4^ Type-II diabetes mellitus (T2DM) accounts for 90% of DM cases, and its prevalence is rapidly increasing in Pakistan. Effective management and treatment adherence are crucial for improving outcomes.^3^,^4^ Treatment adherence is a key factor in the success of pharmacological and non-pharmacological interventions for chronic diseases.

However, non-adherence remains a common challenge, especially in patients with chronic illnesses.^5^,^6^ Studies show adherence rates are lower in developing countries, often falling below 50%.^7^,^8^ Quality of life in diabetics is typically poor due to the burden of complications. Improved glycemic control is associated with better quality of life, and intensive glycemic management does not negatively impact these outcomes.^9^ Effective self-care practices-including healthy nutrition, regular physical activity, glycemic monitoring, proper medication use, and smoking cessation-can help delay complications.^10^,^11^ Healthcare providers play a pivotal role in identifying challenges, promoting self-sufficiency, and encouraging adherence to treatments.^12^ Diabetes education programs have been shown to improve diabetes care and reduce hospitalization rates.^13^-^15^ The Diabetes Control and Complications Trial (DCCT) demonstrated that comprehensive education enhances glycemic control and reduces complications.^15^

In Pakistan, data on medication adherence and quality of life in T2DM patients is limited.^16^ Therefore, this study aimed to evaluate the impact of online diabetes education, provided by International Diabetes Federation (IDF)-certified educators, on adherence and quality of life in patients with T2DM.

## METHOD

This study was a quasi-experimental study conducted in Mardan and its peripheries, Khyber Pakhtunkhwa (KPK), Pakistan. The duration of the study was from October 2022 till March 2023. A total of 360 Patients visited the medical camps conducted for walk in patients, out of which 250 were diabetic while only 196 diabetic patients who fulfilled the inclusion criteria were enrolled in the study using non-probability consecutive sampling technique. Patients with Type-I diabetes, Gestational diabetes, patients with Diabetic ketoacidosis (DKA) and Hyperglycemic Hyperosmolar Non-Ketotic Coma (HONK), patients with complications of diabetes that needed hospitalization were excluded from the study. Data was collected on a pre-designed questionnaire. Demographic information, dietary habits were evaluated based on portion sizes, carbohydrate and fluid intake, and the number of meals consumed per day, treatment regimen for diabetes were asked from every patient and included.

### Ethical Approval:

The ethical approval Ref No:510/LRH/MTI was provided by the Ethical Review Board of lady reading hospital Peshawar.

### Operational instruments:

The medication adherence was assessed by using Morisky Medication Adherence Scale (MMAS) 17 and cause for non-adherence were also inquire. The quality of life was evaluated by Brazilian quality of life.^18^ Patients who scored >8 points on the MMAS were reflected as highly adherent. Patients who scored between 6 to 8 points were considered as medium adherent, and those patients who scored <6 points were considered as low adherent. Brazilian brief version for quality-of-life scale (DQOL-Brazil-8) was used to assess the various domains e.g., level of satisfaction, impact of diabetes, concerns regarding social /vocational and concerns related to diabetes.

Anthropometric and laboratory test such as Body mass index (BMI), blood pressure (BP), Random Blood Sugar (RBS), lipid profile and haemoglobin A1c (HbA1c) were assessed and documented during the first visit to report baseline. After collection of the baseline data, enrolled subjects were counselled by IDF trained diabetes educators regarding healthy lifestyle, diet, and medication. All patients were followed regularly at least twice a month for RBS, BP and medication adherence via telephone call by locally trained personals. HbA1C was also checked and reported at every 12 weeks follow-up in all patients. With timely intervention done wherever needed by the trained diabetes educators. MMAS and DQOL-Brazil-8 were filled from those patients pre and post interventions with a gap of six months to see the overall impact of the interventions.

### Statistical Analysis:

The SPSS software Version 26.0 was used to analyze the data. For numerical data, mean & standard deviation (SD) was determined, whereas for categorical data, frequency and percentages were calculated. The paired t-test was used to compare RBS, HbA1c, BMI, blood pressure, and MMAS scores at baseline and six months after intervention. A statistically significant p-value of less than 0.05 was accepted.

## RESULTS

During the study period, a total of 196 patients were enrolled and 108 (54.6%) patients were male. Almost 165 (84.1%) of the patients had only DM while 31(15.9%) had both DM and hypertension. Family history revealed that 71.3% (119 patients) of the patients had family history of DM and 53 (31.7%) patients had history of hypertension. Family history of DM Type-II was reported in 37(22.2%) patients, while renal and cardiac problems in 14 (8.4%) and 16 (9.6%) patients were observed respectively.

Baseline data regarding their dietary pattern was obtained. Most of the patients preferred to take normal meal 112(58.3%), while 44(22.9%) were taken light meal and 36(18.8%) were taken big meals. More than half of the patients 103(53.6%) preferred both vegetarian and non-vegetarian. Approximately, 133(69.4%) patients were having 3-5 meals, while 58(30.1%) of the patients were consuming 5-6 meals. In addition, 103(52.8%) patients indicated that they drink 1-2 liters of water whereas 85(43.5%) of them were drinking< 1 liter of water.

Treatment protocol revealed that 165 (88.2%) patients were having oral anti-diabetic drugs, 11(5.9%) were using insulin while only 12(6.4%) were taking both. Most frequent reason of non-adherence to medication was financial constrains that was observed in 80(53.3%) patients followed by forget fullness in 62(41.3%) patients and personal reason was indicated by 30(20.0%) patients. On the other hand, excessive use of medication was reported by 17(11.3%) patients. Regarding MMAS score for adherence, almost 66.3% of the patients showed low adherence at first visit. However, medium and high adherence was reported in 32.6% and 1% respectively. The adherence improved significantly at last follow up (after six months) for low, medium and high to 50%, 45.9% and 4% of the patients respectively. The last visit mean MMAS score were improved significantly from first visit with P value < 0.001 as shown in Table-I. The DQOL-Brazil-8 showed improved total satisfaction score at last follow up. Approximately, 25.2% of the patients were very satisfied with their diet and sex life. Similarly, the proportion of individuals suffering from diabetes was decreased after six months. At last, follow up only 5.8% of the patients were worried about the concern regarding diabetes. The other components of DQOL-Brazil-8 and patient’s responses are presented in Table-II.

**Table-I:** Comparison of RBS, HbA1c, BMI, diastolic and systolic blood pressure between baseline and at 06 months follow up.

Variable	Mean ± SD		p-value

	Baseline	6 months follow up	
Random blood sugar (mg/dL)	280.44±105.26	250.97±58.89	<0.001
HbA1c (%)	9.93±2.10	8.98±1.72	<0.001
Body Mass Index(kg/m^2^)	29.61±4.88	27.83±5.48	<0.001
Diastolic Blood Pressure (mm Hg)	89.24±17.24	86.56±5.99	0.069
Systolic Blood Pressure (mm Hg)	141.58±20.84	139.01±14.16	0.026
MMAS score for adherence	4.76±1.95	5.38±1.93	<0.001

RBS: red blood cells; HbA1c: glycated haemoglobin; BMI: Body mass index.

**Table-II:** Brazilian brief version of the Diabetes Quality of Life Measure (DQOL-Brazil-8) during 1^st^ and last visit.

Variables	Very satisfied 1 n (%)	Quite satisfied 2 n (%)	Satisfied 3 n (%)		Little satisfied 4 n (%)		Not at all satisfied 5 n (%)	
***Are you satisfied with the flexibility of your diet?***								
**1^st^ visit**	18(9.1%)	34(17.3%)	52(26.5%)		70(35.7%)		22(11.22%)	
**Last visit**	51(26%)	43(21.9%)	80(40.81%)		16(8.1%)		6(3.06%)	
***Are you satisfied with you sex life?***								
1st visit	30 (15.3%)	36(18.3%)	45(22.9%)		55(28.0%)		30(15.3%)	
Last visit	48 (24.4%)	41(20.9%)	70(35.7%)		26(13.2%)		11(5.6%)	
***Total satisfaction score***								
1st visit	12.24%	17.8%	24.7%		31.8%		13.2%	
Last visit	25..2%	21.42%	38.26%		10.71%		4.33%	
***Impact***								
***Variables***	***Never 1***	***Almost never 2***	***Sometimes 3***		***Almost always 4***		***Always 5***	
***How often do your diabetes interfere with your physical exercises?***								
1st visit	22(11.2%)	23(11.7%)	78(39.7%)		33(16.8%)		40(20.4%)	
Last visit	14(8.3%)	51(30.2%)	81(47.9%)		48(24.4%)		2(1.2%)	
***How often do you eat something that you should not be eating as diabetic patient?***								
1st visit	12(6.1%)	15(7.65%)	66(33.6%)		55(28.06%)		48(24.4%)	
Last visit	20(10.20%)	35(21.0%)	71(42.5%)		38(22.8%)		18(9.1%)	
***How often do you feel bothered about diabetes?***								
1st visit	14(7.14%)	23(11.73%)	66(33.6%)		43(21.9%)		50(25.5%)	
Last visit	67(34.1%)	77(39.28%)	35(17.8%)		14(7.1%)		3(1.5%)	
**Total impact**								
1st visit	8.16%	10.3%	35.7%		22.27%		23.4%	
Last visit	17.17%	27.72%		31.8%		17.00%		3.9%
***Concerns social/vocational***								
***How often do you worry whether you will have children?***								
1st visit	23(11.7%)	26(13.2%)	39(19.8%)		58(29.5%)		50(25.5%)	
Last visit	69(35.2%)	83(42.34%)	51(29.5%)		15(8.7%)		1(0.6%)	
**Concerns related to diabetes**								
***How often do you worry whether you will pass out?***								
1st visit	9(4.59%)	14(7.14%)	40(20.4%)		77(39.2%)		56(28.5%)	
Last visit	49(25.0%)	34(20.0%)	80(40.8%)		27(15.9%)		6(3.6%)	
***How often do you worry whether you will have complications due to your diabetes?***								
1st visit	13(6.63%)	26(13.2%)	78(39.7%)		47(23.9%)		32(16.3%)	
Last visit	25(15.2%)	31(18.9%)	86(43.8%)		37(18.8%)		17(8.6%)	
***Total score***								
1st visit	7.65%	11.22%	26.7%		30.9%		23.46%	
Last visit	18.8%	16.5%	42.3%		16.32%		5.86%	

There was statistically significant difference found between baseline and six months follow up of RBS, Hb1Ac, BMI and systolic and diastolic blood pressure manifesting a p-value of <0.001. All baseline parameters such as RBS, HbA1c, BMI, diastolic and systolic BP and their comparison between baseline and at six months follow up visit are presented in Table-I.

## DISCUSSION

Patients’ education regarding diabetes is the crucial component of the effective management of DM Type-II like any other chronic disease. Diabetes in patients cause complications due to poor perception of illness and insufficient control on blood glucose level.^19^-^21^ The aim of the study was to identify how online structured diabetes education can improve patient’s adherence towards medication as well as quality of life.^22^ Present study reported online diabetes education had a moderate impact on treatment adherence and adherence improved significantly at last follow up (after six months) for low, medium and high to 50%, 45.9% and 4% of the patients respectively while mild impact on quality of life while significant effect on HbA1c, blood glucose level, BMI and BP as at last, follow up only 5.8% of the patients were worrying about the concern regarding diabetes.

Lorig K et al.^22^ evaluated the efficacy of online diabetes self- management program and changes in the HbA1c levels, demonstrated increase exercises and self- efficacy at 6 and 18 months. They have concluded that an online diabetes self- management program is a viable option for patients with Type-II DM as the program may be helpful in reducing HbA1c. However, there was no change in other health or behavioral parameters. In the present study, we found statistically significant differences in the RBS, HbA1c and BMI at six months from the baseline measurements. Several methods have been proposed in the past to self- managed the diabetes through digital health applications.^22^-^25^ Kumar S et al.^23^ evaluate the use of mobile app and its impact on diabetes. This mobile app comprised of in-app coaching by a certified diabetes educator and their impact on glycemic control for patients with Type-II DM. They have reported that this online app significantly controls the glycemic levels and also provided effective self-management education and support to patients. The findings of this study are consistent with our results. We also used somehow, similar system in the presented study through telephonic communication and found significant reduction in HbA1c levels at six months from baseline.

The diabetes education and awareness among patients is a necessary tool. Adequate diabetes education not only improves glycemic index, but also overall health related outcomes and quality of life in individual with Type-II DM.^22^-^25^ It has been observed that the patient education also impacts medication adherence. As it is a well- known fact that medication adherence remains an ongoing problem among Type-II DM patients.^26^ The use of online patient education has also implemented to improve medication adherence and it proved to be an effective method.

The reason of poor adherence may be due to lack of patient education along with other factors such as complex dosing regimens, clinical inertia, safety concerns, socioeconomic issues, ethnicity, social support and polypharmacy.^26^,^27^ Similar findings are reported in our study about the impact of online diabetes education counselling on medication adherence. We found significant difference between baseline and six months follow up score of MMAS for adherence with P- value <0.001. The most prevalent factors for non-adherence to medication was financial constrains that was observed in 80 (53.3%) patients followed by forget fullness in 62 (41.3%) patients and personal reason was indicated by 30 (20%) patients.

### Limitation:

It includes the small sample size which is a major limitation of this study. The lack of control group, recruitment of patients without randomization and small sample size reduces the internal validity of our results. Therefore, we recommend multi-center, randomize control trials on the similar topic to evaluate the effectiveness of online education program in improving the diabetic care. However, our study can serve as a frame work or start point for the future researches on the similar topic.

## CONCLUSION

This study concluded that online diabetes education counseling had a significant impact on treatment adherence. Post intervention quality of life of Type-II diabetics was adequate. Moreover, online diabetes education counseling significantly reduced the blood glucose level, BMI and blood pressure.

### Authors’ Contribution:

**IA:** Conceptualized the idea and responsible for the integrity and accuracy of the study. **AJ** and **NU:** Collected the data and analysed it. **AJ, NU** and **HMK:** Literature search, prepared the first draft of manuscript. **IA** and **HMK:** Revised and approve the final version of manuscript. All authors have read, approved the final version and are accountable for the integrity of the study.
